# Let’s Be Honest: These Medical Malpractice Cases Were a Pain in the Back

**DOI:** 10.5811/cpcem.2022.1.54908

**Published:** 2022-02-28

**Authors:** Samuel Isaias Garcia, Summer Ghiath, Gregory P. Moore, Rachel A. Lindor, Sara Hevesi

**Affiliations:** * Mayo Clinic, Department of Emergency Medicine, Rochester, Minnesota; † Mayo Clinic Alix School of Medicine, Scottsdale, Arizona; ‡ Sandra Day O’Connor College of Law, Tempe, Arizona; § Mayo Clinic, Department of Emergency Medicine, Scottsdale, Arizona

**Keywords:** back pain, cauda equina syndrome, spinal epidural abscess, medicolegal, medical malpractice, documentation honesty

## Abstract

**Introduction:**

This series reviews three cases of back pain where a highly morbid diagnosis was missed by an emergency physician and subsequently successfully litigated.

**Case Report:**

We review the clinical entities of spinal epidural abscess and cauda equina syndrome, challenging diagnoses that can be easily missed and lead to patient harm if not treated promptly. Here we offer suggestions for recognizing these conditions quickly, performing an adequate history and exam, and using documentation to support decision-making.

**Conclusion:**

When confronted with an unfortunate medical outcome, maintaining honesty is of paramount importance in medical-legal environments.

## INTRODUCTION

Back pain is a common complaint seen in the emergency department (ED), and the vast majority of these cases are caused by a benign musculoskeletal etiology. However, there are several rare and serious diagnoses that emergency physicians must consider and treat promptly as they can lead to significant morbidity if missed. Here, we discuss three cases of back pain in which life-threatening causes were initially missed. We discuss the pitfalls and caveats that contributed to these misses and the factors that lead to successful litigation.

### Case 1: Anonymous v Anonymous

Facts: A 26-year-old man presented with lower back pain that was radiating down his legs. He also complained of weakness in his legs. His rectal tone was intact. A screening evaluation was done and his white blood cell count was noted to be elevated. The facility did not have magnetic resonance imaging (MRI), and obtaining this study would have required transfer to another facility two miles away and then a return trip. Thus, the emergency physician ordered a computed tomography (CT) of the lumbar spine, which revealed a possible right iliopsoas abscess. The patient was admitted to the hospital, and over the next 24 hours his condition neurologically deteriorated. He progressed from lower extremity weakness to complete paralysis and inability to breathe on his own. He was transferred to another hospital where an MRI of the cervical spine revealed a cervical spinal epidural abscess (SEA) and he was taken immediately to surgery. The patient remained quadriplegic and required 24-hour skilled care for his lifetime. Plaintiffs litigated, claiming that the delay in diagnosis and treatment resulted in the outcome of paralysis. The lawsuit was settled for $1.98 million.^1^

### Case 2: Wilson v Abington Hospital-Virginia

Facts: A 58-year-old male complained of back pain after raking leaves. He was seen and prescribed percocet. Two days later he was seen again. Radiographs revealed no fracture or evidence of arthritis. No neurologic examination was done. The pain continued for five more days despite his use of percocet. The patient returned and an MRI was ordered that revealed a SEA. He subsequently died from resultant meningitis. The plaintiff brought suit claiming that if the diagnosis would have been considered earlier in the illness, the patient would have survived. The defense claimed that the pain was consistent with a musculoskeletal origin and that further evaluation was not indicated and the care the patient received was within the standard of care. The case went to trial and the jury rendered a verdict for $4.7 million.^2^

### Case 3: Amy Cook and Jason Cook v Betty Agbede

Facts: A 37-year-old woman awoke with severe lower back pain and was unable to stand. She was carried to the car and taken by her husband to the ED. In triage she was greeted by a nurse, and the patient reported severe lower back pain that was radiating down her left leg along with tingling in her left buttocks and groin. The nurse only documented “severe back pain.” She was triaged to the “minor” care part of the ED. A nurse evaluated her, and after the same history reported severe radiating back pain but no mention of tingling. Court testimony revealed that the patient was next seen by a physician and reported the same history but added that it was the worst pain she had ever experienced. The physician did a 2–5 minute evaluation and the only physical examination performed was brushing the front of the shins. The patient was never removed from the wheelchair. The physician described the pain as “moderate,” not radiating, and like similar pain in the past. The physician later said the patient was too calm for it to be severe. The physician also stated that the patient moved herself to the bed, and a full body exam lasting 15 minutes had occurred. The physician claimed the patient denied numbness or tingling. The diagnosis of back strain and chronic back pain was made. The patient was discharged on analgesics. At discharge it was documented that the patient’s pain was 10/10.

The pain persisted, and the next day the patient had difficulty making it to the bathroom before experiencing urinary incontinence. Her primary physician was unavailable, so she made an appointment with a chiropractor two days later. The chiropractor performed radiographs and referred the patient immediately to the ED. In the ED the patient was diagnosed with cauda equina syndrome (CES). Immediate surgery was done, but there was permanent nerve damage resulting in chronic pain, an unsteady gait, and numbness of the groin, leg, and foot, as well as bowel and bladder incontinence.

A lawsuit was brought claiming inadequate evaluation and that the lack of response to analgesics in the ED should have warned the staff of serious pathology and that imaging was indicated. The defense was that given the history and exam there was no reason to suspect the diagnosis of CES or pursue further workup. An investigation of the medical record and electronic footprinting revealed it was impossible that the physician had done a 15-minute exam. This was based on the fact that there was no 15-minute gap in computer entries. A confidential settlement was reached.^3^

## DISCUSSION

### Spinal Epidural Abscess

Spinal epidural abscess is a medical emergency caused by a pyogenic infection in the epidural space. It is known to result in significant spinal cord damage by direct compression, thrombosis, ischemia, or inflammation. Prompt diagnosis and intervention are essential to prevent devastating neurologic compromise, sepsis, and death.^4^ Unfortunately, most cases of SEA have multiple presentations before definitive diagnosis is made. Attorneys covet litigation involving this diagnosis as they can include multiple defendants who had individual opportunities to make the correct diagnosis in a timely fashion.

It is a rare etiology with an incidence of 5.1 cases per 10,000 admissions reported by a single institution.^5^ The mean onset of presentation is at 50 years of age, with the greatest prevalence between 50–70 years. Common risk factors include a history of injection drug use, infective endocarditis, dental abscesses, history of spinal interventions, alcoholism, diabetes, human immunodeficiency virus, and trauma.^6^ The posterior thoracolumbar spine is the region most often involved. The most common causative organisms are staphylococcus aureus, streptococcus, Gram-negative bacilli, and anaerobes.^7^,^8^,^9^

The classic diagnostic triad for SEA is fever, back pain, and neurologic deficits. However, the complete triad is present in only 13–37% of patients.^4^ The most common symptom is back pain, which is seen in 70–100% of the cases. Neurologic deficits such as motor weakness, radiculopathy, and bladder and bowel dysfunction are present in up to 50% of the cases.^10^ Fever is often absent, which may lead to a delayed or missed diagnosis.^11^,^12^ Laboratory evaluation reveals a leukocytosis in only 60% of cases; however, erythrocyte sedimentation rate and C-reactive protein are significantly elevated in nearly all cases of SEA and thus may be more helpful.^10^,^13^,^14^

If a SEA is clinically suspected, imaging of the spinal column should be done emergently as delays in the diagnosis or treatment may worsen the prognosis.^4^ Magnetic resonance imaging is the imaging modality of choice and provides the best localization and extent of inflammation.^15^,^16^,^17^ A CT with intravenous contrast may be a reasonable alternative if an MRI is contraindicated or not available.^18^,^19^ It is important to remember that multiple skip lesions representing several levels of involvement can occur despite patients not having pain in all the affected areas.^10^ This phenomenon impacts many successful malpractice cases as physicians will frequently order an MRI involving only the painful area and the abscess is lurking elsewhere. If a physician is considering this diagnosis it is imperative that an MRI of the entire spine be ordered and not just the area of maximal pain. Once blood cultures are obtained, broad-spectrum antibiotics should be started immediately. Surgical decompression and drainage is the critical emergent treatment of choice for most patients, especially in the presence of acute or progressive neurologic deficits, spinal instability, or ring-enhancing lesions on MRI.^6^,^21^

Spinal epidural abscess is a high-risk area for malpractice litigation. Most patients present to healthcare clinicians multiple times before the diagnosis is finally made.^10^ Failing to make the diagnosis on early visits puts a physician at great risk for a lawsuit. Three large medicolegal databases queried for SEA-related malpractice cases demonstrated plaintiff rulings for approximately 35% (47/135) of all cases, which is significantly higher than ED litigation overall (7.4%). There was an average of $4,291,400 awarded to the plaintiff compared to $816,909 in all of emergency medicine plaintiff awards.^4^,^22^ Previous studies have shown that a delay in the diagnosis, delay in treatment, and presence of neurologic complications are all associated with a significant increase in plaintiff awards.^4^

After assimilating the above information, it is clear why the first two cases above were successfully litigated. In the first case, successful litigation may have been avoided if SEA had been considered in the setting of low back pain, weakness, and leukocytosis. An MRI, if pursued, would have likely revealed the correct diagnosis but only if the entire spine had been examined rather than the lumbar spine alone where the patient was experiencing maximal pain. The physician may have anchored on the CT finding of a possible iliopsoas abscess and thus chose not to pursue the gold standard test (MRI) as it would have taken more effort. Often, successfully litigated lawsuits result from a clinician’s willingness to accept a non-optimal study as an answer to a clinical question. If SEA is clinically suspected, broad-spectrum antibiotics should be initiated and an MRI immediately performed.

In the second case, there was again a successful litigation of SEA as a result of a common pattern in the entity. A clinician must be on guard and suspect SEA especially when there are recurrent visits for severe back pain being labeled as musculoskeletal in nature and should perform a thorough neurologic exam with documentation. Lack of exam/documentation will appear to the jury as an uncaring or less thorough clinician. This became an issue in case 3 as well.

### Cauda Equina Syndrome

Cauda equina is a rare syndrome consisting of one or more of the following: bladder and/or bowel dysfunction; reduced sensation in the saddle area; and sexual dysfunction with possible neurologic deficit in the lower limbs. It is estimated to occur in fewer than 1 in 2000 patients presenting with severe low back pain and is caused by an intraspinal lesion distal to the conus that involves greater than or equal to two of the 18 nerve roots of the cauda equina.^23^ The clinical diagnosis is made through a thorough history and physical exam and is confirmed with radiographic studies. The radiographic modality of choice for CES is an MRI. In situations where an MRI is unobtainable, a CT with myelography can be considered.^24^ Once identified, prompt decompression is recommended.

Potential etiologies include structural causes such as intervertebral disc herniation or tumors, infection such as SEA, inflammatory conditions such as spinal arachnoiditis, chronic inflammatory demyelinating polyneuropathy or sarcoidosis, and iatrogenic causes, among other rare etiologies.^23^ It is essential to identify any hallmark symptoms such as severe low back pain, saddle anesthesia, urinary retention, bowel or bladder dysfunction, or sexual dysfunction. The most common symptoms are severe back pain and radiculopathy seen in 83% and 90% of cases, respectively. It has been reported that most patients have an objective sensory/motor deficit in the lower extremities and approximately 76% have decreased perineal sensation.

Urinary retention is also commonly seen in about 60% of cases and can be evaluated by obtaining a post-void residual bladder volume. Values of more than 100 milliliters should raise suspicion of urinary retention. Urinary incontinence is similarly seen in about 55% of reports. Erectile dysfunction is uncommon, seen in less than 5% of cases on initial presentation, but has been reported to be a poor prognostic symptom. It is important to assess for rectal tone, as decreased tone would further support the diagnosis of CES.^24^ Prompt diagnosis is imperative as failure to diagnose this can result in serious morbidity and increased medicolegal risk.^23^

Even though CES is a rare condition, it has a disproportionately higher frequency of malpractice litigation. This is because patients are often left with a high degree of disability. In a five-year period, Britain’s National Health Service cited 24 malpractice claims where 50% of the cases resulted in damages paid with an average payout of $309,166. The highest settlement was $2,979,860.^25^ In another 2004 study, 48% of finalized cases of CES reported to the Medical Defense Union in the United Kingdom resulted in payment of damages to the claimant with an average settlement of $549,427 where about half of these cases involved an incorrect or delayed diagnosis.^23^ In a third study over five years, 55% of finalized cases of CES reported to the Medical Protection Society resulted in payment of damages to the claimant with an average settlement of $171,303 per case. The highest settlement was $852,640.^25^

### Charting Documentation Errors/Dishonesty

Case 3 illustrates mistakes and inadequacies related to documentation in the patient chart that can increase liability for the physician. Lawsuits related to documentation are common, playing a role in up to 20% of malpractice cases.^26^ One study found that the three most common documentation-related malpractice claims surround missing documentation (70%), inaccurate content (22%), and poor mechanics (18%).^27^ Inaccurate content often arises from using templates that automatically populate a normal physical exam or review of systems, or by documenting information that contradicts what was written by other healthcare clinicians. Cases involving poor mechanics increasingly revolve around transcription errors, resulting in wrong medications or wrong dosages being administered to patients. Lawyers are more likely to pursue litigation when the patient record has low quality documentation as it often provides irrefutable evidence of a mistake.^27^

An anonymous case from Massachusetts provides an example of contradicting patient charts. In this case, a patient presented to the ED with right-sided chest pain and was admitted for pain control after the ED evaluation failed to identify a cause.^28^ In the hospital, the patient decompensated, was found to have a spontaneous, chest wall hematoma causing hemorrhagic shock, and subsequently died. The patient’s family sued the emergency physician for not diagnosing the patient sooner. The physician tried to argue that the patient looked well with no signs of serious illness in the ED, which is what was documented in his physical exam, but the family’s lawyer was able to show that the ED nurse had described the patient as “cool, moist, and mottled.” This conflicting description cast doubt on the veracity of the physician’s exam, and the patient’s family was ultimately awarded $800,000 in damages. This example illustrates the importance of reviewing other clinicians’ notes to find and resolve any potential inconsistencies in the patient chart.

*Perry v. United States* highlights the dangers of altering the medical record.^29^ In this case, a five-week-old was brought into the ED twice within the same day with complaints of a fever. The same physician discharged the patient without a thorough infectious workup. On the third visit, the patient was eventually diagnosed with meningitis but suffered permanent neurologic deficits as a result. During court proceedings, it was found that the physician had changed the chart to remove evidence of the patient’s fever during the initial visits. The court found the physician at fault and issued a $20 million verdict, an amount that likely reflected the dishonesty demonstrated by this attempt at alteration. Some medical malpractice policies will not cover physicians if there is evidence of chart alteration, and in some states such behavior could be grounds for losing licensure and negate any caps on damages that would otherwise be applicable. Electronic health systems keep records of all activities within a patient’s chart, making this type of behavior easy to detect, and the consequences can be profound. Therefore, it is important to not engage in dishonest charting and to avoid inaccuracies in documentation. Evidence of dishonesty or blatant inaccuracies will ultimately lead to the courts finding the physician liable.

In case 3, the patient presented with severe lower back pain with signs of radiculopathy and was unable to stand. The clinician performed an inadequate history and physical examination and failed to recognize red flag symptoms. The clinician was also dishonest in her report of the length of assessment that was done. A delayed diagnosis in this situation resulted in permanent nerve damage resulting in an unsteady gait, urinary and fecal incontinence, and chronic pain.

After realizing the above information, in the third case, successful litigation may have been avoided if a thorough history and physical examination had been performed. In patients presenting with back pain, a full neurologic examination is essential. Key components of the exam include evaluating for saddle anesthesia, sensation, strength, rectal tone, and examining the patient’s gait. Identification of concerning symptoms such as inability to stand, neurologic deficits, and severe pain unresponsive to analgesics should prompt further evaluation. Once CES is suspected, an MRI should be immediately performed.

## CONCLUSION

We have discussed several presentations of back pain and identified the factors that led to a failure to diagnose. In the first case a suboptimal study (CT) was performed, which identified an abnormality thought to be the cause of his symptoms, while missing the diagnosis that ultimately led to significant morbidity. If a spinal epidural abscess is suspected, an MRI of the entire spine should be performed as skip lesions can occur and be found outside the area of maximal pain. In the second case, a patient presented repeatedly with severe back pain requiring opiates for pain control. It is important to remember that repeat visits and severe pain warrant increased scrutiny to ensure the correct diagnosis is not being overlooked and that the care team is not succumbing to anchoring bias. A thorough physical exam would likely have revealed a concerning etiology in both the second and third cases. While prepopulated or templated physical exam notes can be a helpful time saver, they should accurately reflect the examination that was done and not falsify how much time physicians spent with patients. As highlighted by these cases, there is truly no substitute for a thorough history, physical exam, and complete workup in cases of back pain where there are red flag symptoms suggesting a dangerous underlying pathology.

### Take Home Points

- To avoid litigation for spinal epidural abscess and cauda equina syndrome, it should be realized that correct diagnosis is usually made after multiple visits: the key to diagnosis is suspecting it.
- It is imperative to image the entire spine when pursuing the diagnosis of spinal epidural abscess. This is because skip lesions can occur, and abscesses may be found in a separate location other than the site of maximal pain.
- Both spinal epidural abscess and cauda equina syndrome are a favorite litigating diagnosis of malpractice attorneys as they lead to both higher and more frequent awards.
- Clinicians must have a high index of suspicion as spinal epidural abscess and cauda equina often masquerade as musculoskeletal back pain and do not always present in a classic fashion.
- It is very difficult for a medical-legal defendant to be successful in a verdict when it has been discovered that they have been dishonest
